# STERNAL INTERFERENTIAL CURRENT STIMULATION AFTER STERNOTOMY: A RANDOMIZED, SHAM-CONTROLLED TRIAL ON PAIN AND WOUND HEALING

**DOI:** 10.2340/jrm.v57.43941

**Published:** 2025-08-20

**Authors:** Nils SCHULZ, Gian VO, Pascal VAN WIJNEN, Tim WILHELMI, Michael COCH, Uwe LANGE, Philipp KLEMM

**Affiliations:** 1 Department of Rheumatology, Immunology, Osteology and Physical Medicine, Justus-Liebig-University Giessen, Campus Kerckhoff, Kerckhoff Klinik, Bad Nauheim, Germany; 2 Department of Internal Medicine and Cardiology, Median-Klinik am Südpark, Bad Nauheim, Germany; 3 Department of Cardiology, Hochgebirgsklinik Davos, Davos, Switzerland

**Keywords:** cardiac rehabilitation, cytokines, electric stimulation therapy, pain management, postoperative pain, sternotomy, wound healing

## Abstract

**Objective:**

To evaluate the effect and safety of serial interferential current stimulation on postoperative pain and wound healing after sternotomy in cardiac rehabilitation.

**Design:**

Prospective, randomized, double-blinded, sham-controlled clinical trial.

**Subjects/Patients:**

200 patients undergoing open-heart surgery via sternotomy were enrolled during inpatient cardiac rehabilitation, 8 to 12 days postoperatively.

**Methods:**

Patients were randomized into an intervention group receiving interferential current stimulation over the sternum (6 sessions across 21 days) or a control group receiving sham stimulation. All participants underwent the same standardized cardiac rehabilitation programme. The primary outcome was pain reduction over 21 days. Secondary outcomes included analgesic use, inflammatory cytokine levels, pulmonary function, wound healing, and adverse events.

**Results:**

Interferential current stimulation significantly reduced pain scores compared with sham treatment. Analgesic use decreased more in the intervention group. A greater reduction in tumour necrosis factor alpha and interleukin 6 levels was observed. Pulmonary function and quality of life improved in both groups without significant between-group differences. No adverse effects or wound infections occurred in the intervention group.

**Conclusion:**

Serial interferential current stimulation may be a safe, effective non-pharmacological therapy for reducing post-sternotomy pain and analgesic use in cardiac rehabilitation. The effect may be mediated by modulation of inflammatory cytokines.

## LAY ABSTRACT

After heart surgery, many patients suffer from strong chest pain, which makes breathing and moving difficult. This can slow down recovery and increase the risk of complications. While pain medications can help, they often lead to side effects, especially in older adults. In our study, we tested a type of electrical stimulation applied to the chest to relieve pain and support wound healing. We compared this treatment with a group that received no active stimulation. Among 200 patients in rehabilitation, those who received the real stimulation reported significantly less pain and needed fewer pain-relieving medications. We also saw signs of reduced inflammation in the blood. Although all patients had metal wires placed around their sternum during surgery, no complications from the electrical stimulation occurred. This non-drug approach could be a safe and helpful option for managing pain as part of standard rehabilitation after heart surgery.

Following cardiosurgical sternotomy patients frequently experience postoperative oedema, haematoma, and significant sternal pain, particularly during deep inspiration, coughing, or sneezing (1). Shallow breathing and weak cough, which often result from pain and reduced inspiratory effort, lead to retained secretions and atelectasis, creating a favourable environment for pneumonia development (2–4). Incentive spirometry and other forms of physical therapy help to mitigate infection risk and reduce pain (2). To achieve sufficient participation in physical and rehabilitative exercises, effective pain management is crucial. However, the use of analgesics, including nonsteroidal antirheumatic drugs (NSAIDs), paracetamol, metamizole, and opioids is limited by dosage, drug interactions, and individual patient factors like kidney function. Moreover, it is associated with numerous side effects, particularly in multimorbid elderly patients, who make up a large proportion of this patient group. In Germany, for example, over 70% of patients undergoing cardiac surgery are older than 60 years, with 38% aged 70 or above (5). Studies have linked analgesics to increased overall mortality in both the general population and especially patients with cardiovascular diseases (6–9). These limitations and risks underscore the need for non-pharmacological pain management strategies. Therefore, cardiac rehabilitation (cREHA) is recommended in Germany as an evidence-based intervention to improve outcomes following a cardiac event or cardiac surgery (5, 10). Despite its established benefits, challenges remain, particularly in addressing postoperative pain and optimizing recovery in elderly patients.

In recent years, significant progress has been made in physical medicine, particularly in understanding its effects on pain by modulating cytokine activity (11, 12). Electrotherapy, a key component of physical medicine, is routinely employed for pain relief in musculoskeletal disorders, improving circulation, and wound healing, as well as promoting neural regeneration (13–15).

Interferential current stimulation (ICS), operating within 1–100 kHz, offers specific advantages over low-frequency and direct-current therapies, including reduced skin resistance and elimination of tissue burn risk. ICS has demonstrated efficacy in alleviating musculoskeletal pain, reducing oedema, and enhancing post-surgery mobility (16, 17).

We hypothesized that serial ICS is a safe and effective non-pharmacological treatment option reducing postoperative pain following sternotomy, potentially through cytokine modulation, and that it would positively influence functional and patient-reported outcomes within the framework of multimodal cardiac rehabilitation.

## METHODS

### Study design

This was a prospective monocentric, randomized, sham-controlled, double-blinded parallel-group study with a 1:1 allocation comparing serial ICS vs sham ICS over 21 days in patients who had undergone open-heart surgery. Methods and trial design were not commenced after trial start.

### Setting

The study was conducted at the Median-Klinik am Südpark in Bad Nauheim, Germany, a specialized inpatient cardiological rehabilitation centre. Recruitment and data collection took place between 1 February 2013 and 30 December 2015. All participants were enrolled at the start of their inpatient cREHA, typically 8–12 days after sternotomy.

### Participants

Patients undergoing open-heart surgery via sternotomy (e.g., coronary artery bypass grafting, aortic or mitral valve replacement, or reconstruction) and admitted to inpatient cREHA 8–12 days postoperatively were eligible. In addition, patients had to have a pain intensity of at least 30/100 measured by visual analogue scale (VAS). Exclusion criteria included contraindications to serial ICS, such as metal implants, implanted electronic devices (e.g., pacemakers), active wound infections in the treatment area, peripheral arterial occlusive disease stages III and IV, febrile illnesses, and oncological conditions with a risk of metastasis.

It should be noted that, despite the general contraindications for metal implants, all patients had metal or wire cerclage of the sternum following sternotomy.

Participants were randomized in a 1:1 ratio into an intervention group (IG) and a control group (CG).

All patients were thoroughly informed about the study and provided written consent to participate. The study was approved by the Ethics Committee of Justus-Liebig-University Giessen (AZ 204/12) and retrospectively registered prior to analysis in the German Registry of Clinical Studies (DRKS) under the number DRKS00021266.

### Intervention

The IG received serial ICS applied to the sternal region (Nemectron Edit device, dual bipolar electrode, Endosan current form, constant 4 kHz frequency, current intensity 10–20 mA, application duration of 18 minutes, six applications over 21 days; Nemectron GmbH, Karlsruhe, Germany). ICS sessions were administered twice weekly on Tuesdays and Thursdays according to a standardized schedule, with interventions conducted between 08.00 and 14.00 (8.00 a.m. to 2.00 p.m.) throughout the rehabilitation period.

*Dosage of current intensity*. At the beginning of therapy, the current intensity is gradually increased until the patient feels a sensation of current (slight tingling) (sensitive stimulation threshold). This stimulation threshold was maintained for the duration of treatment; in sensitive patients, the application was subthreshold (sensitive subthreshold).

In the CG (sham treatment), the current was reduced and switched off after the sensitive stimulation threshold was reached. The patients were informed that treatment with the sensitive threshold could not be carried out permanently and could be tolerated.

All participants received an identical standardized multimodal cREHA programme over a period of 21 days, including disease-specific education, behavioural therapy, exercise therapy, nutritional counselling, classical massage, heat packs applied to the thoracic spine region, therapeutic breathing exercises, and endurance training.

Primary and secondary outcomes were evaluated at baseline and after completion of the cREHA programme (21 days).

### Primary outcome

*Pain.* The change in pain intensity was measured using the VAS, assessing pain experienced during the last 7 days (0 = no pain, 100 = worst pain). The primary endpoint was the between-group difference in pain intensity change from baseline to day 21.

### Secondary outcomes

*Analgesic usage.* Analgesic consumption was qualitatively and quantitatively recorded, including NSAIDs, paracetamol, metamizole, and opioids from baseline to day 21. The change in analgesic intake was compared between the IG and CG to assess potential analgesic-sparing effects of the intervention.

Cytokine serum levels:

- Tumour-necrosis-factor alpha (TNF-α): measured with the Human TNF-α ELISA Kit (RayBiotech, Inc, Peachtree Corners, GA, USA, Cat#: ELH-TNFalpha-001, range 3.5–600 pg/mL).
- Interleukin-6 (IL-6): measured using the Elecsys IL-6 Test (Roche Diagnostics, Mannheim, Germany), a chemiluminescence immunoassay performed on the Cobas 6000 analyser (range 1.5–500 pg/mL).

The between-group differences in cytokine level changes from baseline to endpoint were evaluated to explore potential anti-inflammatory effects of the intervention.

*Lung function parameters.* Lung function testing was performed using spirometry to measure lung volumes and airflow rates. Lung function parameters (vital capacity [VC] in percentages and forced expiratory volume in 1 s [FEV1] in percentages) were assessed at baseline and at day 21 using the CARDIOVIT CS-200 spirometry system by SCHILLER (Baar, Switzerland), in accordance with the standards of the European Coal and Steel Community (ECSC). Changes in these parameters were compared between groups to examine potential respiratory benefits of the intervention.

*Patient-reported quality of life.* Patient-reported quality of life was assessed at baseline and at day 21 using the HeartQoL health-related quality of life questionnaire. The HeartQoL questionnaire is a validated tool designed to assess health-related quality of life in patients with cardiovascular diseases. It consists of 14 items across 2 subscales (physical and emotional), scored on a 4-point Likert scale, with higher scores indicating greater impairment (18). Changes in HeartQoL scores from baseline to endpoint were compared between the IG and CG.

*Safety and wound healing.* Wound healing and other adverse events were monitored and descriptively recorded throughout the study period. Any relevant differences in complication rates between the groups were explored qualitatively.

### Sample size calculation

The sample size was calculated based on the primary outcome (pain intensity reduction). Assuming a clinically relevant between-group difference of 10 points on the VAS, a standard deviation of 20, a 2-sided α of 0.05, and a power of 90%, the required sample size was 84 patients per group. To allow for potential dropouts and to ensure sufficient power for secondary outcomes, we aimed to enrol 100 patients per group.

### Randomization and allocation

Participants were randomized using computer-generated block randomization with variable block lengths. A total of 41 blocks were used, with block lengths ranging from 2 to 8. Patients were allocated equally (1:1 ratio) to the IG or CG, with 100 participants per group.

### Blinding

The study employed a sham-controlled double-blind design. Neither patients nor assessors were aware of group allocation. Only the individual operating the electrotherapy device was privy to the patient’s allocation to ensure proper sham or active treatment administration.

To support blinding in the sham group, participants were informed that perceptible stimulation might not be maintained continuously due to tolerability and safety considerations. This explanation was not provided to the active treatment group. The ICS device emitted no visible or audible signals, and no direct feedback was accessible to participants.

Although both groups were treated in the same physical therapy rooms, treatments were scheduled individually, and patients received therapy one-on-one with the therapist. While no specific measures were in place to prevent informal communication between participants, the individual treatment format was intended to reduce the risk of treatment contamination.

### Statistical analysis

Continuous variables were summarized using means and standard deviations. Changes between baseline and follow-up (Δ-mean) were calculated for each variable. Normality of distributions was tested using the Shapiro–Wilk test to determine the use of parametric or non-parametric tests.

Analgesic use was descriptively assessed at the beginning and end of the study to objectively evaluate changes over time.

For all continuous outcomes, analysis of covariance (ANCOVA) was conducted with the post-intervention value as the dependent variable. Each model included the respective baseline value as a covariate. Additionally, age, sex, and type of surgery (coronary artery bypass grafting, valve surgery, other) were included as covariates for all models to control for potential confounding. These variables were selected *a priori* based on their known or plausible associations with postoperative recovery and outcome response.

ANCOVA assumptions were assessed: homogeneity of variances was confirmed using Levene’s test; residual normality was evaluated visually with Q–Q plots and tested using the Shapiro–Wilk test. Although minor deviations from normality were observed, ANCOVA was deemed sufficiently robust given the sample size.

Adjusted group differences (IG vs CG) were estimated using marginal means, and are reported with 95% confidence intervals (CI).

Categorical outcomes (e.g., analgesic use) were compared using χ^2^ tests. To examine the odds of analgesic reduction or discontinuation, odds ratios (OR) with 95% CI were calculated using logistic regression.

*P*-values for secondary outcomes were adjusted using the Bonferroni–Holm correction to account for multiple comparisons, mitigating the risk of Type I errors. The primary outcome (pain intensity) was tested without adjustment, as it was based on a single, predefined hypothesis. Statistical significance was defined as *p* < 0.05.

Statistical analyses were performed using R version 4.4.1 for Windows (R Foundation for Statistical Computing, Vienna, Austria).

## RESULTS

**Fig. 1:**
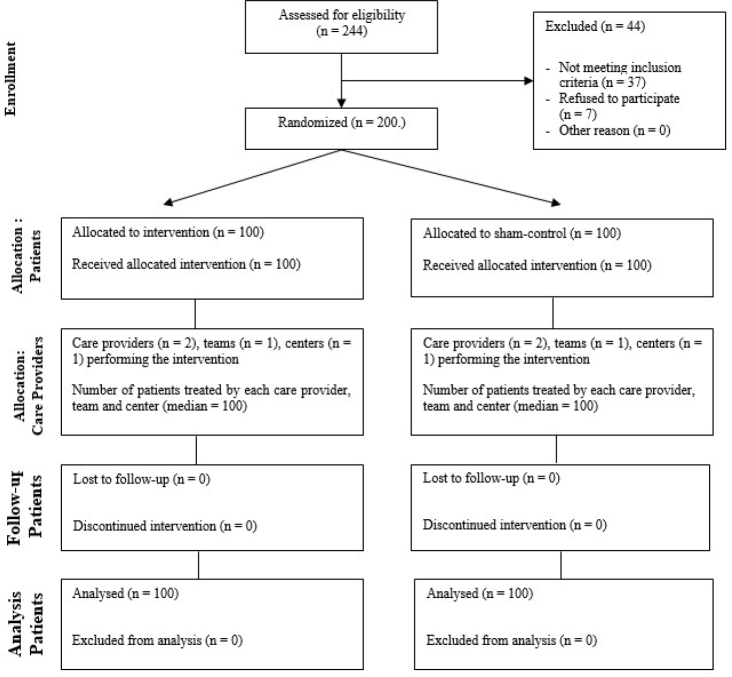
Modified CONSORT flow diagram for individual randomized controlled trials of nonpharmacologic treatments.

A total of 244 patients who underwent sternotomy were screened for the study. Of these, 37 patients (15.2%) did not meet the inclusion criteria or had contraindications for therapy with serial ICS (metal implants, *n* = 9 [3.6%]; implanted pacemakers, *n* = 13 [5.3%]; peripheral arterial occlusive disease stages III and IV, *n* = 15 [6.1%]). Additionally, 7 patients (2.9%) refused to participate. Ultimately, 200 patients were included in the study. All patients completed the study and could be analysed (Fig. 1). The resulting patient characteristics are presented in Table I.

**Table I:** Patient characteristics and laboratory parameters (baseline)

Item	IG (*n* = 100)	CG (*n* = 100)
Age, years, median (SD)	70 (15)	69 (13)
Female	42 (42)	49 (49)
Reason for sternotomy, *n* (%)		
Coronary artery bypass grafting (CABG)	48 (48)	45 (45)
Heart valve surgery	44 (44)	50 (50)
Aortic surgery	4 (4)	2 (2)
Other	4 (4)	3 (3)
Analgesic medication, *n* (%)	88 (88)	91 (91)
Metamizole	35 (35)	26 (26)
NSAID	33 (33)	42 (42)
Opioids	31 (31)	35 (35)
Pain, VAS, median (SD)	47.6 (14.1)	39.7 (10.3)
Metamizole, drops, median (SD)	20.25 (33.30)	12.50 (24.37)
TNF-α, pg/mL, median (SD)	20.5 (6.7)	18.1 (4.4)
IL-6, pg/mL, median (SD)	2.1 (1.6)	1.9 (1.1)
VC, %, median (SD)	57.74 (15.23)	59.41 (14.70)
FEV1, %, median (SD)	62.89 (17.75)	63.81 (16.15)
HeartQoL, median (SD)	2.18 (0.41)	2.23 (0.37)

IG: intervention group; CG: control group; SD: standard deviation; VAS: visual analogue scale; TNF-α: tumour-necrosis-factor alpha; IL-6: interleukin 6; VC: vital capacity; FEV1: forced expiratory volume in 1 s; HeartQoL: heart quality of life questionnaire.

### Pain

**Fig. 2:**
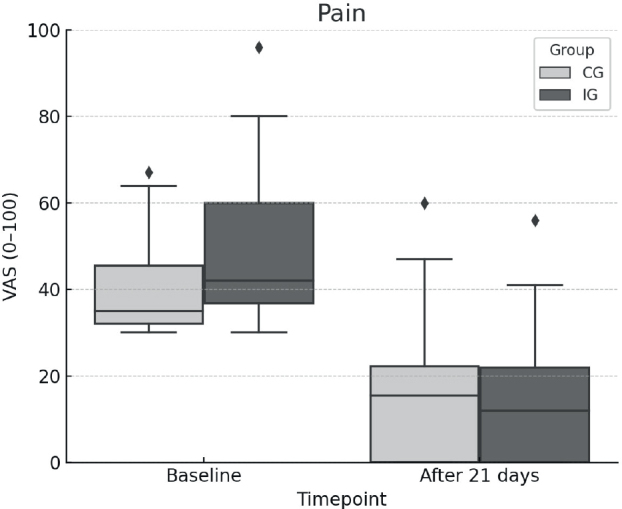
Changes in pain over time. VAS: visual analogue scale; CG: control group; IG: intervention group.

At baseline, the IG reported a pain intensity of 47.6 (standard deviation (SD) 14.1), while the CG reported 39.7 (SD 10.3). At follow-up, the IG reported a mean pain intensity of 13.3 (SD 13.0), while the CG reported 15.6 (SD 14.0). ANCOVA showed an adjusted mean difference of –8.3 (95% CI: [−11.2, −5.4]; *p* < 0.001, Fig. 2) in favour of the IG. The partial η² for the group effect was 0.08, indicating a moderate effect.

**Fig. 3:**
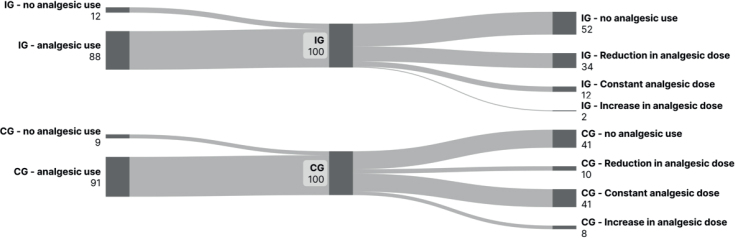
Comparison of changes in analgesic usage between the 2 groups after 21 days. IG: intervention group; CG: control group.

Additionally, a noticeable reduction in analgesic usage was documented in the IG (Fig. 3): At baseline, 88 (88%) patients in the IG and 91 patients (91%) in the CG were on analgesic medication. At the end of the rehabilitation programme, this decreased to 52 cases (52%) in the IG and 41 cases (41%) in the CG. The analgesic dose was reduced in 34 cases (34%) in the IG and 10 cases (10%) in the CG at study end. Medication remained unchanged in 12 cases (12%) in the IG and 41 cases (41%) in the CG. Conversely, an increase in analgesic dose was necessary in only 2 cases (2%) in the IG compared with 8 cases (8%) in the CG.

When combining dose reduction and discontinuation of analgesic medication at the end of the study compared with baseline, a statistically significant difference between the 2 groups in favour of the IG was observed (95% CI: [2.97, 11.74]; *p* < 0.001). The OR was 5.9, with a significantly higher odds of dose reduction or discontinuation in the IG compared with the CG (95% CI: [2.8, 12.4]; *p* < 0.001).

In line with this, a reduction in the number of metamizole drops administered per patient was observed. At baseline, the IG received a mean of 20.3 drops (SD 33.5), while the CG received 12.5 drops (SD 24.5). At follow-up, the mean intake had decreased to 4.3 drops (SD 14.7) in the IG and 6.3 drops (SD 18.6) in the CG. Analysis of covariance, ANCOVA, revealed an adjusted mean difference of −4.91 drops (95% CI: [−8.49, −1.33]; unadjusted *p* = 0.007, Holm-adjusted *p* = 0.037) in favour of the IG. The partial η² for the group effect was 0.021.

### Cytokine serum levels

**Fig. 4:**
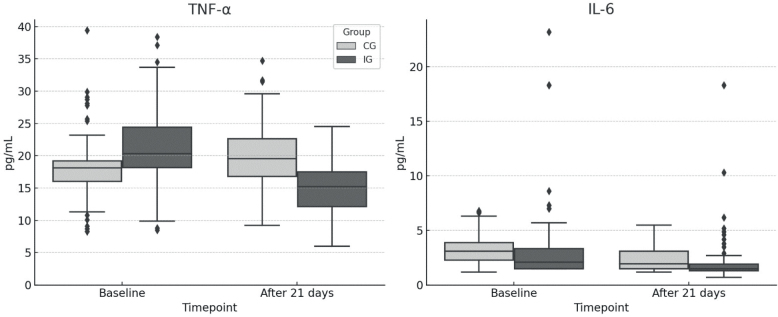
Changes in cytokine serum levels over time. TNF-α: tumour-necrosis-factor alpha; IL-6: interleukin 6; CG: control group; IG: intervention group.

In both groups, reductions in TNF-α and IL-6 serum levels were observed over the course of the study. For TNF-α, mean concentrations decreased from 8.94 pg/mL (SD 5.27) to 2.31 pg/mL (SD 2.28) in the IG, and from 8.83 pg/mL (SD 5.61) to 6.65 pg/mL (SD 4.82) in the CG (Fig. 4). ANCOVA revealed an adjusted mean difference of −7.00 pg/mL (95% CI: [−8.04, −5.97]; unadjusted *p* < 0.0001, Holm-adjusted *p* < 0.0001) in favour of the IG. The partial η² for the group effect was 0.348, indicating a large effect.

For IL-6, mean serum levels decreased from 2.58 pg/mL (SD 2.43) to 1.37 pg/mL (SD 1.42) in the IG, and from 2.48 pg/mL (SD 2.28) to 1.65 pg/mL (SD 1.57) in the CG (see Fig. 4). ANCOVA revealed an adjusted mean difference of −0.36 pg/mL (95% CI: [−0.63, −0.09]; unadjusted *p* = 0.01, Holm-adjusted *p* = 0.04) in favour of the IG. The partial η² for the group effect was 0.012.

### Parameters of lung function

For VC, mean values increased from 63.1% (SD 13.7) to 73.8% (SD 14.5) in the IG and from 62.6% (SD 13.9) to 68.9% (SD 14.8) in the CG. ANCOVA revealed an adjusted mean difference of 3.69% (95% CI: [0.48, 6.90]; unadjusted *p* = 0.024, Holm-adjusted *p* = 0.073) in favour of the IG. The partial η² for the group effect was 0.016.

For FEV1, mean values increased from 65.2% (SD 14.3) to 73.9% (SD 15.4) in the IG and from 64.9% (SD 14.7) to 72.0% (SD 16.0) in the CG. ANCOVA revealed an adjusted mean difference of 1.30% (95% CI: [–2.48, 5.07]; *p* > 0.05).

### Patient-reported quality of life

At baseline, mean HeartQoL scores were comparable between the IG (2.18, SD 0.41) and the CG (2.23, SD 0.37), with both groups showing improvements over the course of the study (IG: 2.60, SD 0.38; CG: 2.66, SD 0.30). The mean change in HeartQoL was 0.42 (SD 0.34) in the IG and 0.43 (SD 0.35) in the CG, representing a clinically significant improvement in both groups. ANCOVA revealed an adjusted mean group difference of −0.039 (95% CI: [−0.118, 0.04]; *p* > 0.05). Similarly, there were no significant differences between the groups in the physical and emotional subdomains of the questionnaire (*p* > 0.05 for both).

### Safety and wound healing

In 2 of the 200 patients (1%), surgical site infections occurred during the course of the cREHA. These were classified as superficial surgical site infections, presenting with redness and swelling but without secretion formation. Both cases were observed in CG without serial ICS. No adverse effects of the current application or surgical site infections were observed in the IG.

## DISCUSSION

To the best of our knowledge, this randomized, sham-controlled trial is the first to investigate whether sternal ICS can effectively reduce postoperative pain and promote wound healing following cardiosurgical sternotomy. In addition, the study examines its impact on analgesic use, cytokine-mediated pain modulation, pulmonary function, wound healing, and patient-reported quality of life.

The application of serially applied sternal ICS was associated with a greater reduction in pain, analgesic use, and serum levels of TNF-α and IL-6 compared with sham stimulation. While trends favouring the IG were observed in the absolute values of VC and FEV1, the differences did not reach statistical significance. An improvement in quality of life, as measured by the HeartQoL questionnaire, could not be demonstrated.

Pain intensity is a known factor affecting rehabilitation success. Studies have shown that high pain levels can limit participation, lead to therapy discontinuation, and result in shorter rehabilitation duration with reduced treatment exposure (19, 20).

While there was a significant reduction in pain during the cREHA programme in both groups, the pain reduction was significantly greater in the IG in which the serially applied sternal ICS was performed. This aligns with prior evidence on the effectiveness of electrotherapy in reducing pain in musculoskeletal and post-surgical contexts (16, 17). Importantly, the reduction in pain was accompanied by a statistically significant decrease in analgesic use, with a sixfold higher odds of dose reduction or discontinuation in the IG compared with the CG (*p* < 0.001; OR 5.9). This is particularly relevant given the well-documented risks of analgesic medications, including increased overall mortality and side effects, especially in elderly multimorbid patients (6–9).

The significant reduction in pain observed in the IG following ICS therapy may be pathophysiologically explained by its modulatory effects on the cytokine profile, particularly the downregulation of proinflammatory mediators. It is well established that cytokines such as TNF-α and IL-6 play a central role in the development and transmission of pain, especially in inflammatory and neuropathic conditions (21). TNF-α acts as a key driver of the inflammatory response by stimulating the release of additional cytokines, including IL-1β and IL-6, and directly enhances the excitability of nociceptive neurons through upregulation of pain receptors such as TRPV1, thereby amplifying pain -perception (22). Experimental studies have demonstrated that pharmacological blockade of TNF-α, for instance with agents such as etanercept or infliximab, reduces mechanical hyperalgesia and macrophage infiltration in dorsal root ganglia (23).

IL-6, similarly, contributes to central sensitization and neuronal hyperexcitability, with expression in both neurons and glial cells. Its effects are often potentiated through binding to its soluble receptor (sIL-6R), further enhancing neuronal sensitivity within the spinal cord (24, 25). Notably, IL-6 has been shown to act synergistically with TNF-α, as IL-6 blockade can significantly attenuate TNF-α–induced hyperalgesia, suggesting a functional interdependence between these 2 cytokines in the modulation of pain (26).

The significant reductions in TNF-α and IL-6 levels observed in the IG (Holm-adjusted *p* < 0.001 and *p* = 0.040, respectively) suggest a robust anti-inflammatory effect of serial ICS. This may offer a plausible mechanistic explanation for the observed pain relief and supports its potential role as a non-pharmacological intervention targeting inflammatory pain mechanisms.

To date, no human clinical study has investigated the effects of interferential current therapy on cytokine levels. Earlier *in vitro* studies using human monocytes and macrophages yielded inconsistent results depending on stimulation parameters (27). Apart from the present findings, similar anti-inflammatory effects have only been reported for direct transcranial electrical stimulation in patients with knee osteoarthritis (28), further underscoring the novelty and potential clinical relevance of ICS as a therapy with immunomodulatory properties.

Despite trends favouring the IG in pulmonary function parameters (VC, FEV1), differences between groups did not reach statistical significance. For VC, the between-group difference was statistically significant in the unadjusted analysis (*p* = 0.024), but lost significance after correction for multiple comparisons using the Holm method (adjusted *p* = 0.073). This may be due to the relatively short study duration and the multifactorial nature of lung function recovery, which is influenced by pre-existing conditions, adherence to breathing exercises, and individual variability (2, 29).

Both groups improved in quality of life, as measured by the HeartQoL questionnaire, which likely reflects the general benefit of structured rehabilitation. Although patients entered the programme with relatively high baseline HeartQoL scores, the observed improvements indicate that the questionnaire was sufficiently sensitive to detect meaningful changes. No additional effect of ICS could be demonstrated in this domain.

Although formally listed as a contraindication for ICS, all patients in this study had sternal wires or cerclages in place. Despite the widespread use of ICS in rehabilitative medicine, only 2 case reports in the literature described severe burns related to ICS application of which only 1 involved a metal implant (30, 31). Moreover, the reported adverse effects were associated with underlying hypoesthesia and improper electrode placement (30, 31). Consistent with these findings, no adverse events occurred in the IG of this study, either in the region of the sternal wires or systemically, indicating good short-term tolerability of ICS in patients with sternal wires or cerclages in place within the monitored setting.

During the course of the study, 2 cases of superficial wound infections were observed, both within the CG that did not receive ICS. This observation supports existing evidence from systematic reviews, which highlight the therapeutic potential of electrical stimulation for wound healing. Pulsed and bidirectional currents, including ICS, have been shown to promote tissue regeneration, accelerate wound healing, and reduce wound size, particularly in complex or postoperative wounds (32, 33).

This study has several strengths that underscore the validity and clinical relevance of its findings. The randomized, double-blinded, sham-controlled design minimized potential biases and ensured the reliability of the observed effects. Additionally, the comprehensive evaluation of outcomes including pain, analgesic use, cytokine levels, lung function, and quality of life provided valuable insights into the multifaceted impact of serial ICS. However, the study also has limitations that should be considered when interpreting the results. Although the observed cytokine reductions may provide a mechanistic basis for the analgesic effects of ICS, causality cannot be established within this study design. Although model assumptions for ANCOVA were formally evaluated and considered acceptable overall, the non-normality of residuals should be noted when interpreting the results. Due to the lack of a follow-up after cREHA, no statements can be made about long-term effects. While the sample size was adequate to detect significant differences in primary outcomes, it may have been underpowered to reveal smaller yet clinically relevant changes in secondary parameters like lung function (VC, FEV1) or patient-reported quality of life (HeartQoL). Moreover, as a single-centre study, the generalizability of the findings is limited, and replication in larger, multicentre trials is needed.

Future studies should evaluate the sustainability of ICS effects over time and assess its utility in specific subgroups. These efforts could help define its role within targeted, multimodal rehabilitation strategies.

In conclusion, serial ICS represents a safe and effective non-pharmacological addition to cREHA in patients undergoing sternotomy. By significantly reducing pain – likely mediated through the downregulation of pro-inflammatory cytokines – and the associated decrease in analgesic use, serial ICS may facilitate a more efficient and well-tolerated postoperative recovery.
